# Supplementary material for: Aguhyper: a hyperledger-based electronic health record management framework
Source: PeerJ Comput Sci. 2024 May 22;10:e2060. doi: 10.7717/peerj-cs.2060 (PMC11157618; doi:10.7717/peerj-cs.2060)
Supplement: Supplemental Information 1 [file peerj-cs-10-2060-s001.zip › Codes/model.cto.docx]

/*

* Licensed under the Apache License, Version 2.0 (the "License");

* you may not use this file except in compliance with the License.

* You may obtain a copy of the License at

*

* http://www.apache.org/licenses/LICENSE-2.0

*

* Unless required by applicable law or agreed to in writing, software

* distributed under the License is distributed on an "AS IS" BASIS,

* WITHOUT WARRANTIES OR CONDITIONS OF ANY KIND, either express or implied.

* See the License for the specific language governing permissions and

* limitations under the License.

*/

/**

* Write your model definitions here

*/

namespace aguhyper.network

participant Patient identified by patientId {

o String patientId

}

participant Doctor identified by doctorId {

o String doctorId

o String affiliation

o String situation

}

participant Lab identified by labId {

o String labId

o String affiliation

}

participant Nurse identified by nurseId {

o String nurseId

o String affiliation

}

participant Researcher identified by researcherId {

o String researcherId

o String affiliation

o String situation

}

transaction patientCreation{

o String patientId

}

transaction doctorCreation{

o String doctorId

o String affiliation

o String situation

}

transaction researcherCreation{

o String researcherId

o String affiliation

o String situation

}

transaction nurseCreation{

o String nurseId

o String affiliation

}

transaction labCreation{

o String labId

o String affiliation

}

asset PatientData identified by patientDataId {

o String patientDataId

o String description

o String hash

--> Patient owner

}

transaction assetCreation{

o String patientDataId

o String description

o String hash

o String a

--> Patient owner

}

event Notification{

o String notification

}

transaction DataSharingDoctor{

o String patientId

o String doctorId

o String dataId

--> Patient patient

--> Doctor doctor

--> PatientData data

}

transaction DataSharingResearcher{

o String patientId

o String researcherId

o String dataId

--> Patient patient

--> Researcher researcher

--> PatientData data

}

transaction AnalysisResult{

--> Patient patient

--> Researcher researcher

--> PatientData data

o String result

}
