# Supplementary material for: Aguhyper: a hyperledger-based electronic health record management framework
Source: PeerJ Comput Sci. 2024 May 22;10:e2060. doi: 10.7717/peerj-cs.2060 (PMC11157618; doi:10.7717/peerj-cs.2060)
Supplement: Supplemental Information 1 [file peerj-cs-10-2060-s001.zip › Codes/DataCreation.js]

/* getParticipantRegistry getFactory getAssetRegistry */

/*

* Sample transaction processor function.

* @param {aguhyper.network.assetCreation} x The sample transaction instance.

* @transaction

*/

async function assetCreation(x){

const DataRegistry= await getAssetRegistry('aguhyper.network.PatientData');

var data=getFactory().newResource('aguhyper.network', 'PatientData', x.patientDataId);

var relation = getFactory().newRelationship('aguhyper.network', 'Patient', x.a);

data.patientDataId=x.patientDataId;

data.description = x.description;

data.hash = x.hash;

data.owner=relation;

await DataRegistry.add(data);

}
