# Supplementary material for: Aguhyper: a hyperledger-based electronic health record management framework
Source: PeerJ Comput Sci. 2024 May 22;10:e2060. doi: 10.7717/peerj-cs.2060 (PMC11157618; doi:10.7717/peerj-cs.2060)
Supplement: Supplemental Information 1 [file peerj-cs-10-2060-s001.zip › Codes/DataSharingResearcher.js]

/* getParticipantRegistry getFactory getTransactionRegistry */

/*

* Sample transaction processor function.

* @param {aguhyper.network.DataSharingResearcher} tx The sample transaction instance.

* @transaction

*/

async function DataSharingResearcher(tx) {

const researcherRegistry = await getParticipantRegistry('aguhyper.network.Researcher');

const researcher = await researcherRegistry.get(tx.researcherId);

const patientRegistry = await getParticipantRegistry('aguhyper.network.Patient');

const patient = await patientRegistry.get(tx.patientId);

const assetRegistry = await getAssetRegistry('aguhyper.network.PatientData');

const asset = await assetRegistry.get(tx.dataId);

researcher.situation=tx.dataId+tx.patientId;

// Update the asset in the registry

await researcherRegistry.update(researcher);

var event=getFactory().newEvent('aguhyper.network', 'Notification');

event.notification=asset.hash+ "hash shared with Researcher";

emit(event);

}
