# Supplementary material for: Aguhyper: a hyperledger-based electronic health record management framework
Source: PeerJ Comput Sci. 2024 May 22;10:e2060. doi: 10.7717/peerj-cs.2060 (PMC11157618; doi:10.7717/peerj-cs.2060)
Supplement: Supplemental Information 1 [file peerj-cs-10-2060-s001.zip › Codes/permissions.acl.docx]

/*

* Licensed under the Apache License, Version 2.0 (the "License");

* you may not use this file except in compliance with the License.

* You may obtain a copy of the License at

*

* http://www.apache.org/licenses/LICENSE-2.0

*

* Unless required by applicable law or agreed to in writing, software

* distributed under the License is distributed on an "AS IS" BASIS,

* WITHOUT WARRANTIES OR CONDITIONS OF ANY KIND, either express or implied.

* See the License for the specific language governing permissions and

* limitations under the License.

*/

/**

* Sample access control list.

*/

rule SystemACL {

description: "System ACL to permit all access"

participant: "org.hyperledger.composer.system.Participant"

operation: ALL

resource: "org.hyperledger.composer.system.**"

action: ALLOW}

rule NetworkAdminUser {

description: "Grant business network administrators full access to user resources"

participant: "org.hyperledger.composer.system.NetworkAdmin"

operation: ALL

resource: "**"

action: ALLOW

}

rule NetworkAdminSystem {

description: "Grant business network administrators full access to system resources"

participant: "org.hyperledger.composer.system.NetworkAdmin"

operation: ALL

resource: "org.hyperledger.composer.system.**"

action: ALLOW

}

rule PatientsCanReadDoctorInformations {

description: "Allow all participants read access to all resources"

participant: "aguhyper.network.Patient"

operation: READ

resource: "aguhyper.network.Doctor"

action: ALLOW

}

rule PatientsCanReadResearcherInformations {

description: "Allow all participants read access to all resources"

participant: "aguhyper.network.Patient"

operation: READ

resource: "aguhyper.network.Researcher"

action: ALLOW

}

rule PatientsHasFullAccessToTheirAssets {

description: "Allow all participants full access to their assets"

participant(p): "aguhyper.network.Patient"

operation: ALL

resource(r): "aguhyper.network.PatientData"

condition: (r.patient.getIdentifier() === p.getIdentifier())

action: ALLOW

}

rule PatientsCanReadDataSharingDoctor{

description: "Allow all participants read access to all resources"

participant: "aguhyper.network.Patient"

operation: READ

resource(r): "aguhyper.network.DataSharingDoctor"

condition: (r.patient.getIdentifier() === p.getIdentifier())

action: ALLOW

}

rule PatientsCanReadDataSharingResearcher{

description: "Allow all participants read access to all resources"

participant: "aguhyper.network.Patient"

operation: READ

resource(r): "aguhyper.network.DataSharingResearcher"

condition: (r.patient.getIdentifier() === p.getIdentifier())

action: ALLOW

}

rule PatientHasFullAccessToTheirAssets {

description: "Allow all participants full access to their assets"

participant(p): "aguhyper.network.Patient"

operation: ALL

resource(r): "aguhyper.network.PatientData"

condition: (r.owner.getIdentifier() === p.getIdentifier())

action: ALLOW

}

rule ResearcherCanReadHash{

description: "SCN can read dataUrl when sent money"

participant(p): "aguhyper.network.Researcher"

operation: READ

resource(r): "aguhyper.network.DataSharingResearcher"

condition: (p.situation == r.dataId+r.researcherId)

action: ALLOW

}

rule DoctorCanReadHash{

description: "SCN can read dataUrl when sent money"

participant(p): "aguhyper.network.Doctor"

operation: READ

resource(r): "aguhyper.network.DataSharingDoctor"

condition: (p.situation == r.dataId+r.doctorId)

action: ALLOW

}
