# Supplementary material for: Aguhyper: a hyperledger-based electronic health record management framework
Source: PeerJ Comput Sci. 2024 May 22;10:e2060. doi: 10.7717/peerj-cs.2060 (PMC11157618; doi:10.7717/peerj-cs.2060)
Supplement: Supplemental Information 1 [file peerj-cs-10-2060-s001.zip › Codes/ParticipantCreation.js]

/* getParticipantRegistry getFactory */

/*

* Sample transaction processor function.

* @param {aguhyper.network.patientCreation} x The sample transaction instance.

* @transaction

*/

async function patientCreation(x){

const patientRegistry= await getParticipantRegistry('aguhyper.network.Patient');

var patient=getFactory().newResource('aguhyper.network', 'Patient', x.patientId);

patient.patientId=x.patientId;

await patientRegistry.add(patient);

}

/* getParticipantRegistry getFactory */

/*

* Sample transaction processor function.

* @param {aguhyper.network.doctorCreation} x The sample transaction instance.

* @transaction

*/

async function doctorCreation(x){

const doctorRegistry= await getParticipantRegistry('aguhyper.network.Doctor');

var doctor=getFactory().newResource('aguhyper.network', 'Doctor', x.doctorId);

doctor.doctorId=x.doctorId;

doctor.affiliation=x.affiliation;

doctor.situation=x.situation;

await doctorRegistry.add(doctor);

}

/* getParticipantRegistry getFactory */

/*

* Sample transaction processor function.

* @param {aguhyper.network.labCreation} x The sample transaction instance.

* @transaction

*/

async function labCreation(x){

const labRegistry= await getParticipantRegistry('aguhyper.network.Lab');

var lab=getFactory().newResource('aguhyper.network', 'Lab', x.labId);

lab.labId=x.labId;

lab.affiliation=x.affiliation;

await labRegistry.add(lab);

}

/* getParticipantRegistry getFactory */

/*

* Sample transaction processor function.

* @param {aguhyper.network.nurseCreation} x The sample transaction instance.

* @transaction

*/

async function nurseCreation(x){

const nurseRegistry= await getParticipantRegistry('aguhyper.network.Nurse');

var nurse=getFactory().newResource('aguhyper.network', 'Nurse', x.nurseId);

nurse.nurseId=x.nurseId;

nurse.affiliation=x.affiliation;

await nurseRegistry.add(nurse);

}

/* getParticipantRegistry getFactory */

/*

* Sample transaction processor function.

* @param {aguhyper.network.researcherCreation} x The sample transaction instance.

* @transaction

*/

async function researcherCreation(x){

const researcherRegistry= await getParticipantRegistry('aguhyper.network.Researcher');

var researcher=getFactory().newResource('aguhyper.network', 'Researcher', x.researcherId);

researcher.researcherId=x.researcherId;

researcher.affiliation=x.affiliation;

researcher.situation=x.situation;

await researcherRegistry.add(researcher);

}
